# Diversity, Pathogenicity, and Biological Characteristics of Root Rot Pathogens from *Lycium barbarum* L. in Qinghai Province, China

**DOI:** 10.3390/jof12010062

**Published:** 2026-01-13

**Authors:** Yongbao Zhao, Lingshan Wang, Kaifu Zheng, Chengwen Zheng, Lijie Liu, Hexing Qi

**Affiliations:** 1 College of Agriculture and Animal Husbandry, Qinghai University, Xining 810016, China; 2 Qinghai University, Xining 810016, China

**Keywords:** Gouqi, root rot, *Fusarium* fungi, diversity, pathogenicity, biological characteristics

## Abstract

*Lycium barbarum* L. is an important economic crop in Qinghai province, China. However, root rot seriously reduces the economic results of *L. barbarum*. Here, we collected the diseased *L. barbarum* roots from Nuomuhong Farm of Haixi Mongolian and Tibetan Autonomous Prefecture, Qinghai Province, China, to clarify the diversity, pathogenicity, and biological characteristics of its root rot pathogens. A total of 125 isolates were collected, and based on morphological characteristics and rDNA ITS, *TEF*-*1α*, and *RPB2* genes sequence analysis, they were identified as *Fusarium equiseti*, *F. avenaceum*, *F. solani*, *F. citri*, *F. acuminatum*, *F. culmorum*, *F. sambucinum*, *F. incarnatum*, *F. oxysporum*, *F. tricinctum*, *Microdochium bolleyi*, and *Clonostachys rosea*. These fungi were used to inoculate the roots of 1-year-old *L. barbarum* seedlings using scratching and root-irrigation inoculation methods, and all isolates caused root rot. This is the first report that *M. bolleyi*, *F. avenaceum*, and *F. citri* caused root rot in *L. barbarum*. And the best media, the lethal temperatures, and the optimum carbon sources and nitrogen sources of the 12 pathogen species were determined in this study. Moreover, our findings provide a theoretical foundation for root rot management in the future.

## 1. Introduction

“Gouqi” is the common name for *Lycium barbarum* L., a medicinal plant with a long history of use in traditional Chinese medicine [1]. Chinese medicine makes use of the root bark (Digupi) and fruit (Gouqizi); moreover, the fruit has been widely used as a popular functional food [2,3]. Modern studies have found that *L. barbarum* also has the effects of anti-aging, anti-cancer, and anti-fatigue [4,5], due to the rich content of compounds such as polysaccharides, flavonoid polyphenols, carotenoids, and alkaloids [6,7]. Therefore, the demand for Gouqi and its price are increasing in China. However, the development of the *L. barbarum* industry has been seriously restricted by root rot disease.

Root rot is one of the most common diseases affecting *L. barbarum*, seriously affecting its quality and yield. In the early stages of the disease, the roots have light brown lesions and rot. Roots rot intensifies in the middle stage and the plants have a small amount of defoliation. In the later stage, the rotted roots show reddish brown and the plants shed many leaves until they wither and die [8]. The existence of root rot disease causes a decrease in economic income and seriously affects the development of the Gouqi berry industry in Qinghai Province. At present, there is no effective way of controlling the root rot disease of *L. barbarum*. Meanwhile, the pathogens are soil-borne, which makes their prevention and control even more difficult. Hence root rot in *L. barbarum* is difficult to eradicate and has been called a plant cancer [9].

*Fusarium* species have been reported as the main pathogens causing root rot in *L. barbarum* [10]. *Fusarium acuminatum*, *F. oxysporum*, *F. solani*, *F. concolor*, *F. moniliforme*, *F. equiseti*, and *F. incarnatum* can cause *L. barbarum* root rot disease [11]. *Fusarium* species are *among* the most widely distributed fungi [12]. They are recognized as one of the most important pathogens in agriculture, horticulture, and the food industry [13,14]. They cause economic losses by limiting crop growth and yield or pose a threat to food safety and human health by producing fungal toxins, such as trichothecene, fumonisins, and zearalenone [15,16,17,18]. *Fusarium* species cause many root rots. For example, *Fusarium acuminatum* can cause root rot in *Medicago sativa* [19]; *Fusarium avenaceum*, *F. oxysporum*, and *F. solani* cause root rot in *Pisum sativum* [20]. *Fusarium oxysporum* causes root rot in *Beta vulgaris* [21]. *Fusarium equiseti* causes root rot in *Vigna unguiculata* [22]. *Fusarium incarnatum* causes root rot in *Morus alba* [23], and *F. citri* causes root rot in *Fragaria* × *ananassa* [24]. Moreover, *Fusarium* species interact with other plant pathogens, and mycotoxin accumulation plays an important role in disease outbreaks [25].

The diversity, pathogenicity, and biological characteristics of root rot pathogens from *L. barbarum* in Qinghai Province, China has not been systematically studied. At present, a few studies on *L. barbarum* root rot have been reported, and these mostly focused on the occurrence status of *L. barbarum* root rot and preventive measures [26]. Moreover, we hypothesized that there might be other pathogens besides *Fusarium* species that could infect *L. barbarum*. Therefore, this study sampled root-rotted *L. barbarum* from Nuomuhong Farm in Qinghai province. The objectives of this study were (i) to analyze phylogenetic diversity and morphological characteristics of root rot pathogens from *L. barbarum*; (ii) to determine the pathogenicity of all the isolates to *L. barbarum*; and (iii) to clarify their biological characteristics to provide a foundation for comprehensive disease control.

## 2. Materials and Methods

### 2.1. Collection Site

Gouqi root rot samples were collected from Nuomuhong Farm (96°14′59.95″–96°30′37.46″ E, 36°24′20.21″–36°26′45.66″ N), Dulan County, Haixi Mongolian and Tibetan Autonomous Prefecture, Qinghai Province, China, at an altitude of 2800 m. The area covers 91.3 km^2^, with a low annual rainfall, strong solar radiation, and a short frost-free period [27]. In this study, 36 *L. barbarum* samples showing root rot were collected in July and August 2022 and 2023 from 12 sites on the farm.

### 2.2. Media Used in the Study

The method for preparing the potato dextrose agar (PDA) and 2% water agar (2% WA) medium can be seen in the study by Pašakinskienė et al. [25]. The method for preparing the carrot agar (CA) medium, carnation leaf-piece agar (CLA) medium, complete (CM) medium, mung bean soup culture (MB) medium, and Czapek (Czapek–Dox) medium can be seen in the studies by Zhao et al. [28] and Zhao et al. [29].

### 2.3. Isolation, Purification, and Preservation of Pathogenic Fungi

Root rotted pieces (approximately 0.5 cm) were obtained from the junction of infected and healthy tissues. Then, we used 3% NaClO for 1.5 min and washed 3 times with sterile water. The pieces were dried and placed on 2% WA plates in an incubator at 25 °C. After 2 days, abundant conidiophores and conidia formed on the surface of the roots. Conidia were placed into sterile water with a pipette to prepare a spore suspension. Then, 200 μL of the spore suspension was spread on the 2% WA plate at 25 °C for 12 h. Single spores were selected and cultured on PDA. Fungal isolates were cultured on PDA at 25 °C, maintained on filter paper, and stored at −20 °C for long-term conservation [30].

### 2.4. Morphological Identification

Colony and conidia morphology were observed, and isolates were identified according to the *Fusarium* Laboratory Manual [31]. The mycelial block with a diameter of 5 mm was placed on a PDA plate. After 7 days of culture the diameter was measured using the cross method. Each isolate was tested three times. Conidia were used to make temporary slides, and conidial morphology was observed under a 40× optical microscope (OLYMPUS IX71, Olympus, Tokyo, Japan). The determination method of the length and width of conidia refers to Qi et al. [32].

### 2.5. DNA Extraction, PCR, and Sequencing

Genomic DNA was extracted from vegetative mycelia using the cetyltrimethylammonium bromide protocol [33]. DNA amplification was performed for the DNA sequences of the nuclear ribosomal internal transcribed spacer (rDNA ITS), the translation elongation factor 1-alpha (*TEF*-*1α*), and the RNA polymerase II gene (*RPB2*). The following PCR primers were used: rDNA ITS amplification primers, ITS1 (5′-TCCGTAGGTGAACCTGCGG-3′), and ITS4 (5′-TCCTCCGCTTATTGATATGC-3′) [34,35]; *TEF*-1α gene amplification primers, EF-1 (5′-ATGGGTAAGGARGACAAGAC-3′), and EF-2 (5′-GGARGTACCAGTSATCATGTT-3′) [36,37]; and *RPB2* gene amplification primers, RPB2-5F (5′-GAYGAYMGWGATCAYTTYGG-3′), and RPB2-7CR (5′-CCCATRGCTTGYTTRCCCAT-3′) [38]. DNA sequencing was performed at Tsingke Biological Technology Company (Beijing, China).

### 2.6. Phylogenetic Analysis

The phylogenetic trees were constructed with reference to Crous et al. [39]. DNA sequences were aligned by the MAFFT 7 (https://mafft.cbrc.jp/alignment/software/ accessed on 2 April 2025). Maximum parsimony trees were constructed using IQ-TREE 3.0.1 (http://iqtree.cibiv.univie.ac.at accessed on 10 December 2025) [40], with SH-aLRT support [41], aBayes support [42], and ultrafast bootstrapping (UFBoot2) [43] for estimation of branch support. Branches with SH-aLRT ≥ 80%, aBayes ≥ 0.95, or UFBoot ≥ 95% were considered well supported and were presented in the phylogenetic trees in this order. The combined alignment included *Nectria dematiosa CBS* 127383 as the outgroup. The sequences of the 46 reference strains were downloaded from the NCBI database. The sequences of 125 pathogens were submitted to NCBI GenBank and the accession numbers were PV592419–PV592543 for the ITS sequences, PV567778–PV567888 for *TEF*-*1α*, and PV595151–PV595275 for *RPB2*.

### 2.7. Pathogenicity Assays

The pathogenicity test was performed by inoculating the roots of 1-year-old *L. barbarum* seedlings with conidial suspensions at a concentration of 1 × 10^6^ per mL in 0.025% Tween 20. Two inoculation methods, scratching and root-irrigation inoculation [44], were used to determine the pathogenicity of isolates to *L. barbarum*. For the scratching inoculation method, the root epidermis was evenly pierced with a sterile inoculation needle, and a 10 μL conidial suspension was used to inoculate each wound. Inoculated roots were incubated in a humid and dark chamber at 25 °C for 14 days. For the root-irrigation inoculation method, the *L. barbarum* root epidermis was pierced, and a 15 mL spore suspension was poured into each pot to irrigate the roots. Inoculated plants were incubated in a humid and dark chamber at 25 °C for 24 h and moved to a greenhouse with a 12 h light/12 h dark photoperiod for 30 days. Sterile water was used as a control. The pathogenicity test was repeated three times for each isolate.

### 2.8. Biological Characteristics of Pathogens

The biological characteristics include the optimal media, pH, temperature, carbon and nitrogen sources, and other biological characteristics [28]. And here, the diameter of the isolates was measured by the cross method.

#### 2.8.1. Effects of Different Media

PDA, CA, CLA, CM, and CB media [29] were used to investigate their effects on mycelial growth. A 5 mm diameter mycelial plug was transferred to the center of each medium and then placed into an incubator for 7 days.

#### 2.8.2. Effects of Temperature

To determine the effect of temperature on mycelial growth, 5 mm diameter mycelial plugs of the isolates were incubated at seven temperature gradients (35, 40, 50, 55, 60, 65, and 70 °C) in water bath for 10 min [45]. Then, they were placed into an incubator for 7 days.

#### 2.8.3. Effects of pH

To determine the effect of pH on mycelial growth, PDA media were adjusted with 0.1 M HCl and 0.1 M NaOH to obtain pH values of 4.0, 5.0, 6.0, 7.0, 8.0, 9.0, 10.0, and 11.0 [45,46]. A 5 mm diameter plug was placed in the center of a PDA plate and in an incubator for 7 days.

#### 2.8.4. Effects of Carbon and Nitrogen

Czapek–Dox medium was used as the basic medium to determine the utilization of nitrogen and carbon sources [28]. In the media, 20 g of sucrose was replaced with 20 g of soluble starch, maltose, glucose, dextrin, cellulose, mannitol, lactose, fructose, and xylose to determine the effects of different carbon sources on growth. Furthermore, 4 g of sodium nitrate was replaced with 4 g of beef extract, peptone, yeast extract, urea, potassium nitrate, ammonium nitrate, ammonium chloride, and sodium nitrate to determine the effects of different nitrogen sources on growth. A 5 mm diameter mycelial plug was transferred to the center of each carbon source medium and nitrogen source medium, and plates were incubated at 25 °C in an incubator for 7 days. All experiments were repeated three times.

### 2.9. Data Processing

A Microsoft Excel worksheet was used for data integration; IBM Statistics SPSS 20.0 (SPSS Inc., Chicago, IL, USA) software was used for one-way ANOVA. Shapiro–Wilk tests (*n* < 50) and Kolmogorov–Smirnov tests (*n* < 50) should be performed first to verify data normality. If not, the data needs to be transformed to make it normal. Then, we conducted the one-way ANOVA; different treatments were set as the dependent variable, while the average length of the colony was regarded as the independent variable. Duncan’s multiple range test was used to test the significance of differences (*p* < 0.05).

## 3. Results

### 3.1. Field Symptoms of Gouqi Root Rot

**Figure 1:**
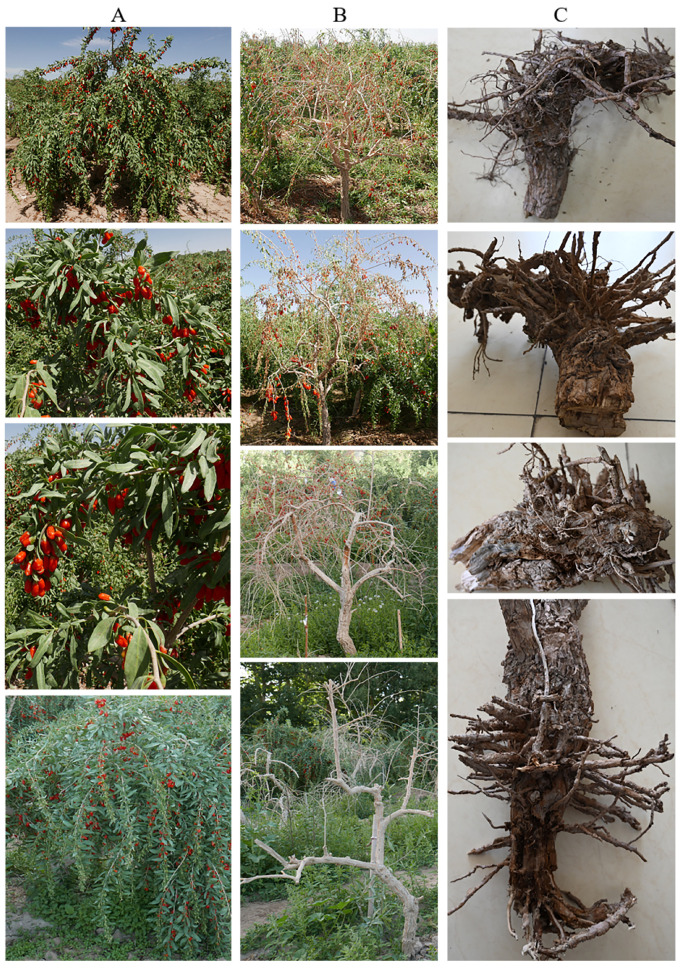
Symptoms of root rot disease on *L. barbarum* in the field: (**A**), healthy Gouqi; (**B**), diseased Gouqi; and (**C**), rotted roots of Gouqi.

The incidence of Gouqi root rot at Nuomuhong Farm, Dulan County, Haixi Mongolian and Tibetan Autonomous Prefecture, Qinghai Province, China, was 10–50%. The leaves on the ground were yellow, and some of the Gouqi leaves fell off. The epidermis near the roots fell off, and the branches and main stems turned white and dry (Figure 1B). The main roots of the underground part were short, and the number of roots was reduced. The fibrous roots were short and thin, and main and fibrous roots were rotted (Figure 1C).

### 3.2. Phylogenetic and Morphology Analysis of Root Rot Pathogens

A total of 125 pathogenic fungi were isolated from 36 root-rotted samples of cultivated Gouqi and identified using morphological and molecular biology techniques. The molecular biological identification of all the pathogens was performed by multilocus sequence analyses of rDNA ITS, *TEF*-*1α*, and *RPB2* genes. After comparison with the reference sequences from NCBI, we found 125 pathogens belong to three genera, namely *Fusarium*, *Clonostachys*, and *Microdochium*.

**Figure 2:**
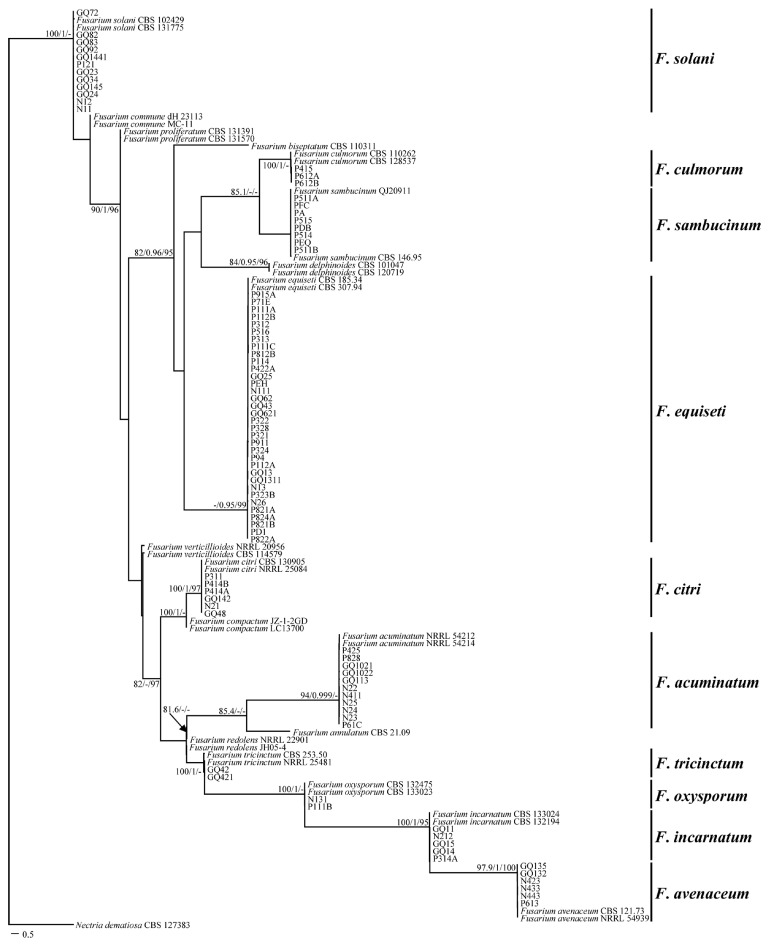
Maximum likelihood tree inferred from combined rDNA ITS, *TEF*-*1α*, and *RPB2* sequences alignment of members of *Fusarium* species from Gouqi. Numbers at the branches indicate support values (SH-aLRT (%) ≥ 80, aBayes ≥ 0.95, Ultrafast bootstrap (%) ≥ 95). The scale bar indicates expected changes per site.

**Figure 3:**
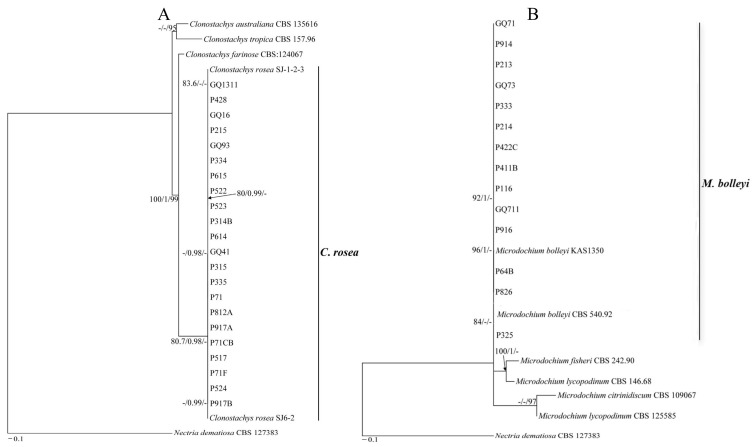
Maximum likelihood trees of root rot pathogens from Gouqi. (**A**) was the maximum likelihood tree inferred from combined rDNA ITS, *TEF*-*1α*, and *RPB2* sequences alignment of members of *Clonostachys* species. (**B**) was the maximum likelihood tree inferred from combined rDNA ITS, and *RPB2* sequences alignment of members of *Microdochium* species. Numbers at the branches indicate support values (SH-aLRT (%) ≥ 80, aBayes ≥ 0.95, Ultrafast bootstrap (%) ≥ 95). The scale bar indicates expected changes per site.

The maximum likelihood trees of *Fusarium* and *Clonostachys* were generated from the combined sequences of rDNA ITS, *TEF*-*1α*, and *RPB2* genes (Figure 2 and Figure 3A), and *Microdochium* were generated from the combined sequences of rDNA ITS and *RPB2* genes (Figure 3B), as the *TEF*-*1α* gene of *M. bolleyi* was not amplified using the primers used in this study. A total of 34 *Fusarium* strains, five *Clonostachys* strains, and six *Microdochium* strains published in the NCBI database were used as reference strains. Overall, the trees with high support values were resolved in the phylogenies inferred for *F. equiseti*, *F. solani*, *F. acuminatum*, *F. sambucinum*, *F. avenaceum*, *F. citri*, *F. incarnatum*, *F. culmorum*, *F. oxysporum*, and *F. tricinctum*, *C. rosea*, and *M. Bolleyi*.

In total, 12 isolates and two control strains (CBS 131775 and CBS 102429) clustered in the *F. solani* clade. P612A, P415, P612B, and two control strains (CBS 110260 and CBS 128527) clustered in the *F. culmorum* clade. Eight isolates and two control strains (QJ 20911 and CBS 146.95) clustered in the *F. sambucinum* clade. Thirty-four isolates and two control strain (CBS 185.34 and CBS 307.94) clustered in the *F. equiseti* clade. Six isolates and two control strains (CBS 130905 and NRRL 25084) clustered in the *F. citri* clade. Eleven isolates and 2 control strains (NRRL 54212 and NRRL 54214) clustered in the *F. acuminatum* clade. GQ42 and GQ421 with control isolates CBS 253.50 and NRRL 225481 in the *F. tricinctum* clade. N131 and P111B in the *F. oxysporum* clade. Five isolates and two control strains (CBS 132194 and CBS 133024) clustered in the *F. incarnatum* clade. Six isolates and two control strains (CBS 121.73 and NRRL 54939) clustered in the *F. avenaceum* clade (Figure 2). Twenty-two isolates and 2 control strains (SJ-1-2-3 and SJ6-2) clustered in the *C. rosea* clade (Figure 3A). Fourteen isolates and two control strains (CBS 540.92 and KAS1350) clustered in the *M. bolleyi* clade (Figure 3B).

**Figure 4:**
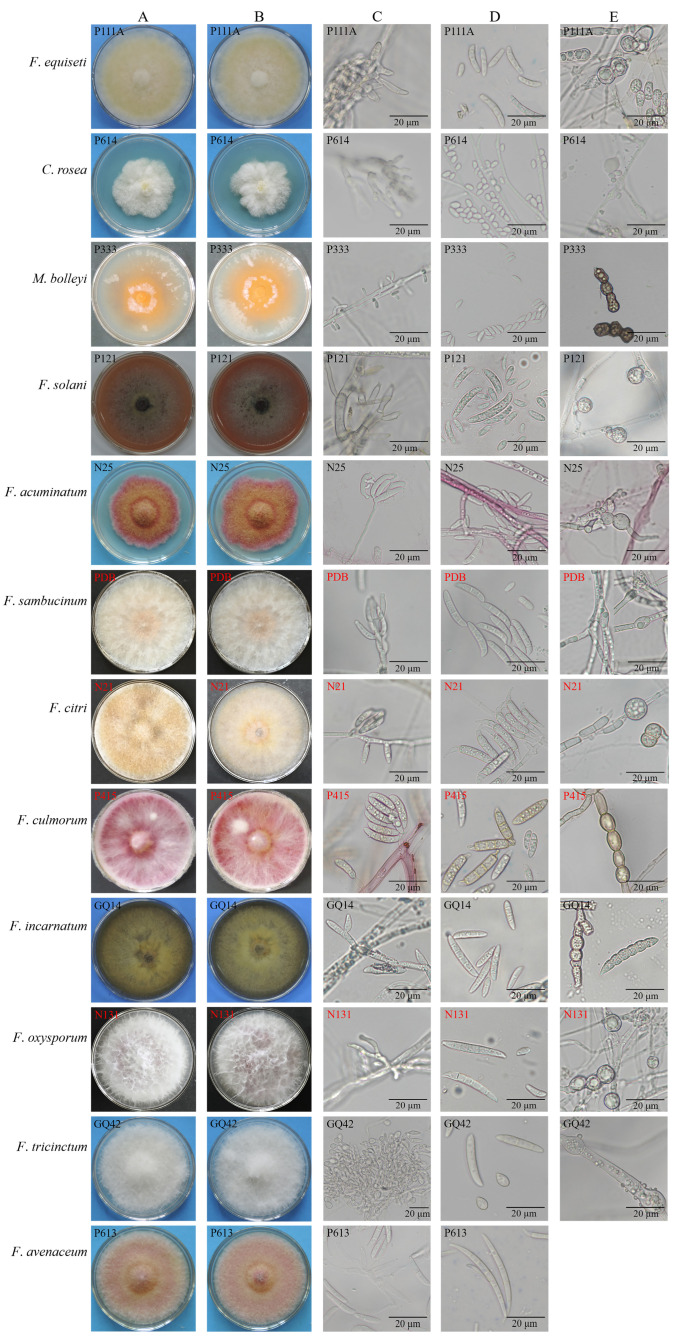
Morphological characteristics of root rot pathogens of Gouqi: (**A**), colony morphology of original isolates; (**B**), colony morphology of re-isolated isolates from infected roots after artificial inoculation; (**C**), conidiophores; (**D**), conidia; and (**E**), chlamydospores.

The morphological characteristics of the 125 pathogens were observed (Figure 4; Table S1). Their chlamydospores were round or ovoid, single or concatenated; however, chlamydospores of *F. avenaceum* were not found. *Fusarium equiseti* produced pale yellow colony; microconidia were sparse and Macroconidia were slightly curved with 1–5 septa. *Clonostachys rosea* formed a white mold, and conidiophores were broom-shaped; conidia were colorless, round or oval, no septum. *Microdochium bolleyi* produced few aerial mycelia, and the middle of the colony was orange; it produced a large number of conidia that were colorless and slightly curved; conidia were produced laterally on the conidiophore. The sparce mycelia of *F. solani* were white in the early stage and produced red, purple, or blue pigments in the later growth stage; macroconidia were colorless, with 1–4 septa; microconidia were ovate to round and single-spored. *Fusarium acuminatum* produced abundant, dense aerial hypha that was rose red; macroconidia were colorless and falciform, had 1–3 septa; microconidia were hyaline, had 0–1 septum. The mycelia of *F. sambucinum* were loose, and was light orange near the center; macroconidia were fascicled, had 1–4 septa; microconidia were colorless, had 0–1 septum. *Fusarium citri* grew abundant, dense pale-yellow mycelia; its macroconidia were colorless and laterally on conidiophores, with 1–5 septa, and the tip of macroconidia was curved; microconidia were rare. *Fusarium incarnatum* had dense hyphae, and the surface was dark yellow; macroconidia were colorless and had 1–5 septa; microconidia were rare. The aerial hypha of *F. culmorum* was sparse, and the colony was red; macroconidia were pale yellow or colorless, with 1–4 septa; microconidia were colorless, ovoid, and had 0–2 septa. The aerial hyphae of *F. oxysporum* were penniform; the colony was light purple in the middle; microconidia were rich, colorless, and had 0–1 septum; macroconidia were colorless, and had 1–4 septa. *Fusarium tricinctum* had white flocculent mycelia; its macroconidia were colorless, falciform, and slightly curved, with 1–5 septa; microconidia were limoniform, oval, or pyriform, with 0–1 septum. The aerial hyphae of *F. avenaceum* were villous and pale red, and macroconidia were thin at both ends and wide in the middle, and had 1–7 septa.

In conclusion, there were 34 *F. equiseti*, 22 *C. rosea*, 14 *M. bolleyi*, 12 *F. solani*, 11 *F. acuminatum*, 8 *F. sambucinum*, 6 *F. avenaceum*, 6 *F. citri*, 5 *F. incarnatum*, 3 *F. culmorum*, 2 *F. oxysporum*, and 2 *F. tricinctum* isolates based on morphological and molecular biological identification, with separation frequencies of 27.2, 17.6, 11.2, 9.6, 8.8, 6.4, 4.8, 4.8, 4.0, 2.4, 1.6, and 1.6%, respectively. Among them, *F. equiseti*, *C. rosea*, and *M. bolleyi* were the dominant pathogens, and *F. avenaceum*, *F. citri*, *F. incarnatum*, *F. culmorum*, *F. oxysporum*, and *F. tricinctum* were rare.

### 3.3. Pathogenicity Analysis

**Figure 5:**
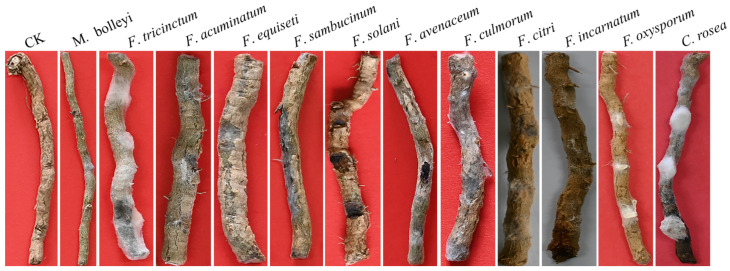
Root rot symptoms of Gouqi using the scratching inoculation method.

The pathogenicity test was conducted using scratching and root-irrigation inoculation methods. The results of scratching inoculation showed that all isolates caused root rot in *L. barbarum* (Figure 5). Light brown spots appeared on the roots after inoculation with pathogens after 3 days, and 7 days later, rots occurred. Notably, distinct black lesions were observed after inoculation with *F. solani*, *F. avenaceum*, and *F. citri*. In contrast, the control group displayed lighter coloration with no symptoms. Robust mycelial growth was visible, with discoloration and decay around the wound, following inoculation with *F. tricinctum*, *F. oxysporum*, and *C. rosea*. Inoculation with *F. oxysporum*, *F. equiseti*, and *F. sambucinum* resulted in significant root decay and a color change at the inoculation site, confirming the disease. Inoculation with *M. bolleyi* and *F. culmorum* led to color changes and rot at the inoculation site.

**Figure 6:**
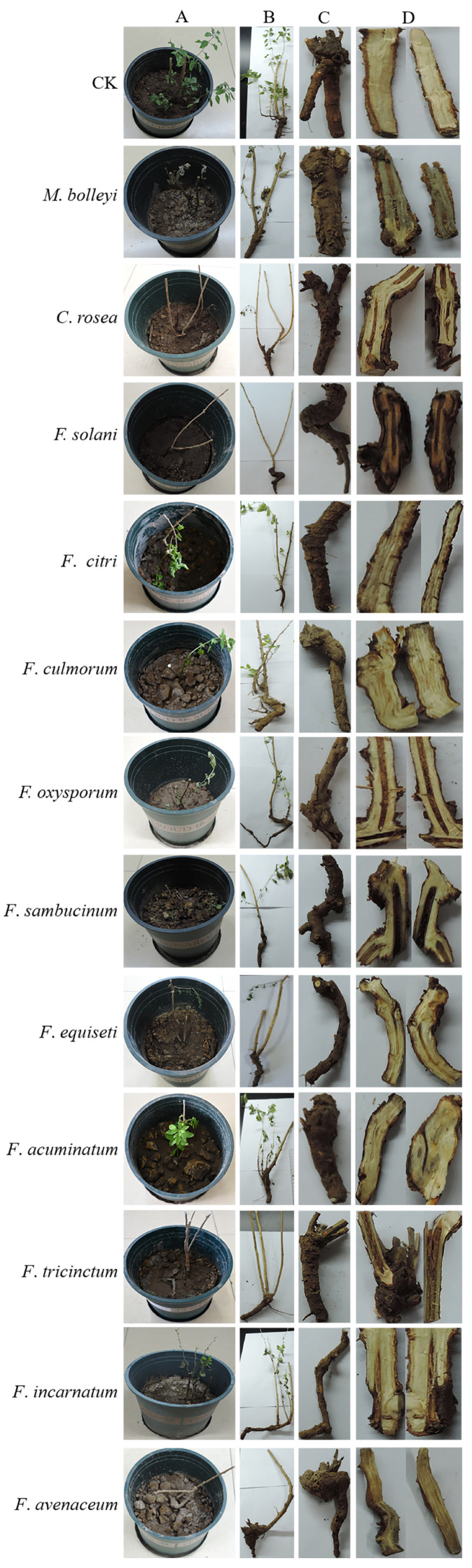
Root rot symptoms of Gouqi using the root-irrigation inoculation method. (**A**), aboveground part; (**B**), inoculated seedlings; (**C**), underground part; and (**D**), root cross-section.

The results of root-irrigation inoculation showed that all isolates could cause root rot (Figure 6). Discoloration and wilting occurred about fifteen days after inoculation; then, the leaves gradually fell off. Thirty days after inoculation, the plants exhibited stunted growth and drying symptoms (Figure 6A,B). Root rot was evident, with internal discoloration visible upon cutting, and black lesions appeared centrally. In contrast, the blank control roots showed no decay or discoloration, maintaining a milky white appearance. Inoculation with *F. solani*, *F. oxysporum*, *F. sambucinum*, and *F. avenaceum* resulted in pronounced internal root discoloration, with the blackened areas larger than those observed after inoculation with other pathogens (Figure 6C,D). Pathogens were re-isolated from the diseased roots, and their morphology matched that of the field pathogens (Figure 4A,B).

### 3.4. Biological Characteristics of Root Rot Pathogens

#### 3.4.1. Effect of Different Media on Mycelial Growth

**Figure 7:**
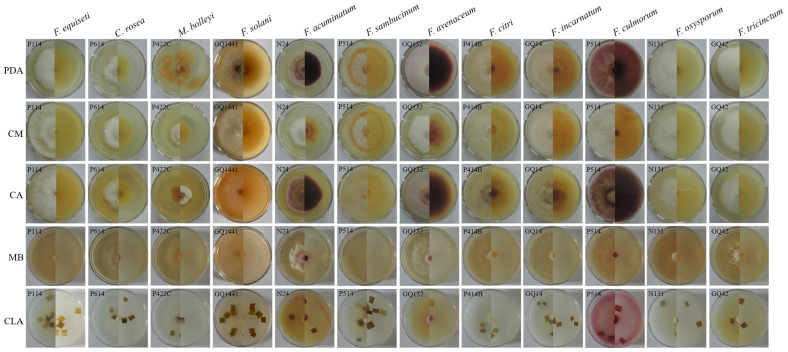
Effects of different media on the pathogen growth rate.

The optimum culture medium for the 12 pathogen species was determined using PDA, CM, MB, CLA, and CA media. The results showed that the best media were PDA, CM, and CA based on the growth condition and growth rate of mycelium. The growth rate of several pathogens on CM and CLA media was the fastest, but the mycelia were only a thin layer and grew poorly. *Microdochium bolleyi*, *Fusarium solani*, *F. sambucinum*, *F. avenaceum*, *F. citri*, *F. incarnatum*, *F. culmorum, F. oxysporum*, and *F. tricinctum* grew well on PDA, with growth rates ranging from 0.47 to 0.80 cm/d. *Fusarium equiseti*, *C. rosea*, *F. sambucinum*, *F. citri*, *F. incarnatum*, and *F. culmorum* grew well on CM, with growth rates ranging from 0.50 to 0.80 cm/d. *Fusarium acuminatum*, *F. sambucinum*, *F. incarnatum*, and *F. culmorum* grew well on CA, with growth rates ranging from 0.62 to 0.80 cm/d (Figure 7, Table S2).

#### 3.4.2. Effect of Temperature on Mycelial Growth

**Figure 8:**
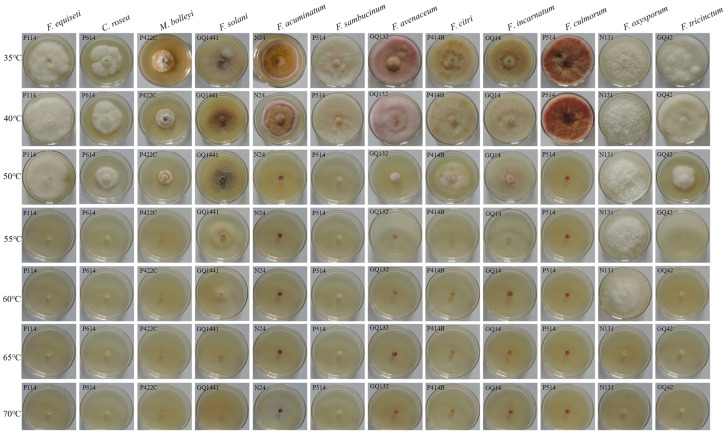
Effects of different temperatures on the pathogen growth rate.

The lethal temperatures of the 12 pathogen species were between 50 and 65 °C. The lethal temperatures were 50 °C for *F. acuminatum*, *F. sambucinum*, and *F. culmorum*; 55 °C for *F. equiseti*, *C. rosea*, *M. bolleyi*, *F. avenaceum*, *F. citri*, *F. incarnatum*, and *F. tricinctum*; and 65 °C for *F. solani* and *F. oxysporum* (Figure 8, Table S3).

#### 3.4.3. Effect of pH on Mycelial Growth

**Figure 9:**
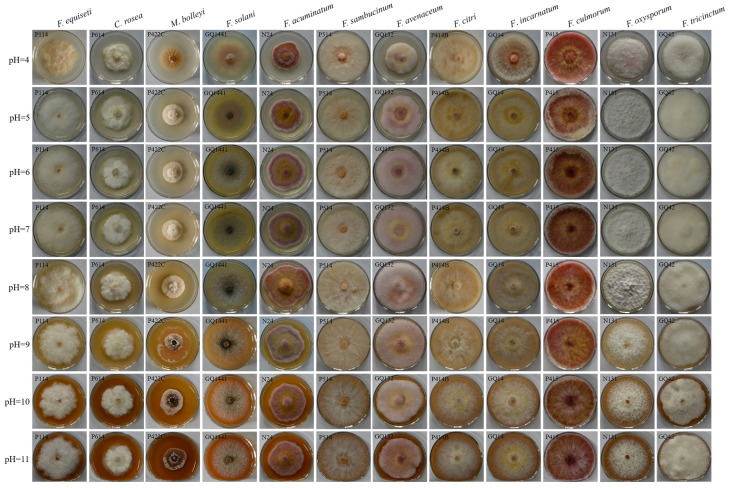
Effects of different pH values on the pathogen growth rate.

The optimum pH of the 12 pathogen species ranged from 4 to 11. The optimum pH of *C. rosea*, *F. citri*, *F. oxysporum*, *F. sambucinum*, *F. equiseti*, and *F. tricinctum* was 4–7, with growth rates ranging from 0.52 ± 0.02 to 0.80 ± 0.01 cm/d. The optimum pH of *F. incarnatum*, *F. culmorum*, *C. rosea*, *F. solani*, *F. citri*, *F. oxysporum*, *F. sambucinum*, *F. equiseti*, *F. tricinctum*, and *F. avenaceum* was 7–11, with growth rates from 0.53 ± 0.03 to 0.80 ± 0.01 cm/d (Figure 9, Table S4).

#### 3.4.4. Effect of Carbon and Nitrogen Sources on Mycelial Growth

**Figure 10:**
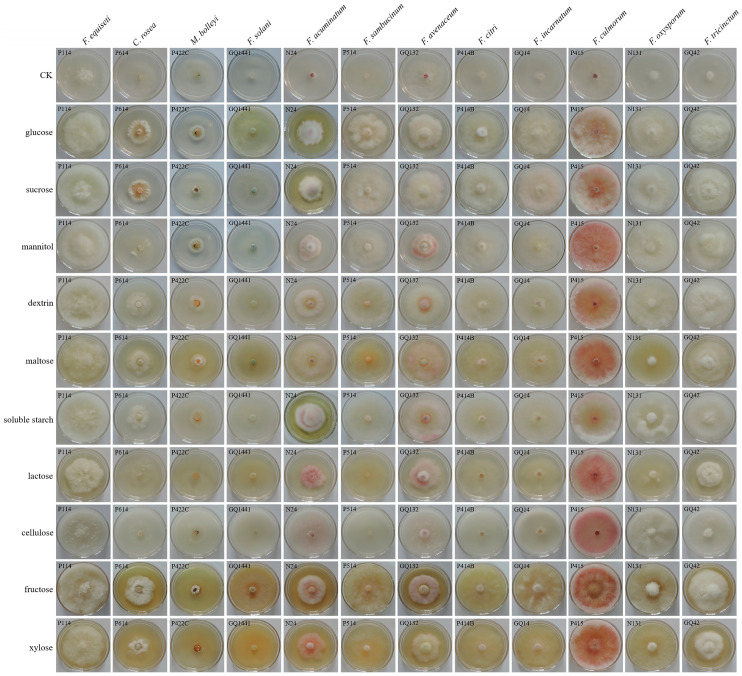
Effects of different carbon sources on the pathogen growth rate.

The optimum carbon sources for the 12 pathogen species were screened using 10 carbon sources. The optimum carbon source for *F. equiseti*, *F. citri*, *F. incarnatum*, *F. culmorum*, and *F. oxysporum* was sucrose, and the growth rates of all were 0.80 cm/d. The optimum carbon source for *C. rosea* was fructose, with growth rates of 0.57 ± 0.03 cm/d. Dextrin was the optimum carbon source for *F. incarnatum*, *F. culmorum*, *F. oxysporum*, and *F. tricinctum* with growth rates of 0.80 cm/d. The optimum carbon source for *F. acuminatum* was cellulose, showing a growth rate of 0.72 ± 0.03 cm/d. Soluble starch was the optimum carbon source for *F. avenaceum*, *F. incarnatum*, *F. culmorum*, and *F. oxysporum*, resulting in growth rates of 0.76 ± 0.0, 0.80 ± 0.01, 0.80 ± 0.01, and 0.80 ± 0.01 cm/d, respectively. The growth rates of CK for *F. equiseti*, *F. incarnatum*, *F. culmorum*, and *F. oxysporum* were also fast, but the mycelia were only a thin layer and grew poorly (Figure 10, Table S5).

**Figure 11:**
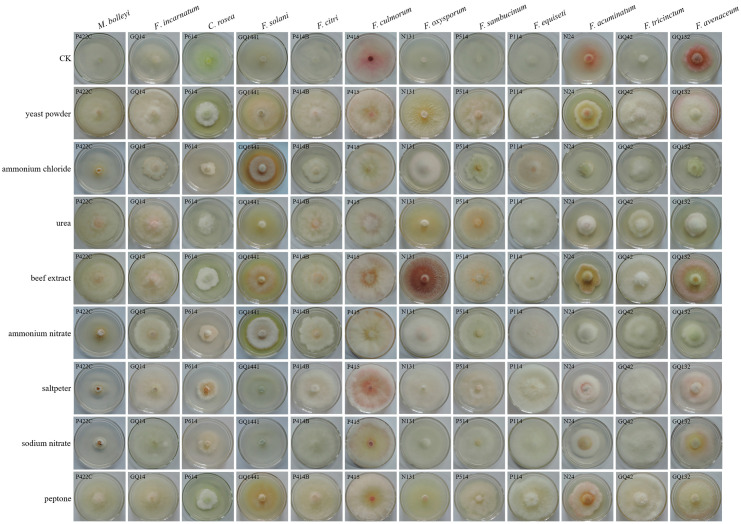
Effects of different nitrogen sources on the pathogen growth rate.

The optimum nitrogen sources for the 12 pathogen species were determined using 8 nitrogen sources, and we found that beef extract, peptone, and yeast powder resulted in optimum growth. The optimum nitrogen source for *F. oxysporum*, *F. tricinctum*, *F. equiseti*, *M. bolleyi*, *F. sambucinum*, *F. incarnatum*, *and F. culmorum* was beef extract, showing growth rates of 0.80 cm/d. Peptone was the optimum nitrogen source for *F. equiseti*, *C. rosea*, *M. bolleyi*, *F. incarnatum*, *F. culmorum*, *F. oxysporum*, and *F. tricinctum*. Yeast powder was the optimum nitrogen source for *F. equiseti*, *M. bolleyi*, *F. sambucinum*, *F. avenaceum*, *F. citri*, *F. incarnatum*, *F. culmorum*, *F. oxysporum*, and *F. tricinctum*, with growth rates ranging from 0.78 to 0.80 cm/d. Saltpeter was the optimum nitrogen source for *F. equiseti* and *C. rosea*, with growth rates 0.80 ± 0.01 and 0.51 ± 0.06 cm/d, respectively. The optimum nitrogen source for *F. solani* was sodium nitrate, with a growth rate of 0.79 ± 0.01 cm/d. The growth rates of CK for *F. equiseti*, *F. acuminatum*, *F. culmorum*, and *F. oxysporum* were fast; however, the mycelia were sparse and only a thin layer (Figure 11, Table S6).

## 4. Discussion

Root rot is one of the main reasons for the decrease in *L. barbarum* yield, and it occurs from the seedling stage to the mature plant stage [47]. Root rot pathogens of *L*. *barbarum* mainly damage its root and rhizome, resulting in reduced water, nutrient transport, and plant death [48]. Nuomuhong Farm is one of the main *L. barbarum*-producing areas in Qinghai Province, China. The large-scale cultivation of *L. barbarum* has brought huge economic benefits. In the first half of 2025, the export volume of Gouqi berries reached 14.792 million yuan in Qinghai; the products were sold to more than 30 countries and regions around the world, and the export volume accounted for over 90% of the total Gouqi berry exports in China [49]. However, root rot is becoming increasingly serious in *L. barbarum*. To effectively control root rot in *L. barbarum*, it is necessary to systematically identify the pathogens causing this disease.

### 4.1. This Is the First Report of M. bolleyi, F. avenaceum, and F. citri Causing Root Rot of L. barbarum in China

In this study, 125 pathogens isolated from rotted roots of *L. barbarum* were confirmed as pathogens based on the results of scratching and root-irrigation inoculation. Morphological and molecular biological identification revealed that these pathogens belonged to 12 species. Among the pathogens, *F. equiseti*, *C. rosea*, and *M. bolleyi* were dominant. *Fusarium avenaceum*, *F. citri*, *F. incarnatum*, *F. culmorum*, *F. oxysporum*, and *F. tricinctum* were isolated less frequently. Additionally, *F. sambucinum*, *F. acuminatum*, and *F. solani* were common species. To our knowledge, this is the first report of *M. bolleyi*, *F. avenaceum*, and *F. citri* as pathogens causing root rot of *L. barbarum* in China.

### 4.2. Fusarium Species, M. bolleyi, and C. rosea Are Important Phytopathogens

In China, *Fusarium* can affect other medicinal plants, such as *Angelica* [50] and *A. membranaceu* var. *mongholicus* [32]; Other plants, such as *Solanum lycopersicum* [51], *Codonopsis pilosula*, and *Zea mays*, also suffer from root and stem rot due to this pathogen; furthermore, *Fusarium* causes needle blight in coniferous trees, such as *Pinus thunbergii* [52]. Abroad, *Fusarium* can lead to root rot and wilt in many important economic and ecological plants, including *Cucumis melo* [53], *C. sativus* [54], and *P. pinea* [55]. *Microdochium bolleyi* typically resides endophytically in plant roots [25]. Some studies have shown that *M. bolleyi* causes crown and root rot in grasses, such as *T. aestivum* and *H. vulgare* [56], and is associated with basal rot of *Agrostis stolonifera* [57] and root rot in *Linum usitatissimum* [58]. *Clonostachys rosea* is a potent biocontrol agent against *F. oxysporum*, using mycoparasitism and its bioactive metabolites [59]. However, *C. rosea* can also cause root rot of *Glycine max* [60], naked *H. vulgare* var. *nudum*, *Xanthoceras sorbifolium* [61], *Polygonatum cyrtonema Hua* [62], *Allium sativum* [63], and *Astragalus membranaceus* [64]. Therefore, we will pay more attention to root rot caused by *Fusarium* species, *M. bolleyi,* and *C. rosea* in the future.

### 4.3. The Composition of Root Rot Pathogens from L. barbarum Varies in Different Regions of China

*Fusarium oxysporum*, *F. solani*, *F. tricinctum*, and *F. chlamydosporum* and *A. alternata* are responsible for root rot disease in Gouqi plants in Gansu and Ningxia provinces, China [11]. *Fusarium culmorum* and *F. equiseti* can cause root rot on *L. barbarum* in Qinghai Province, China [47]. Pathogens from Gouqi roots in the Qaidam Basin, China, were mainly *Fusarium* species, including *F. lichenicola*, *F. oxysporum*, *F. redolens*, and *F. solani*; moreover, *Plectosphaerella plurivora*, *Ple. cucumerina*, *Mortierella alpina*, *Mucor hiemalis*, *Penicillium janthinellum*, *Verticillium nonalfalfae*, *C. rosea*, and *Pen. simplicissimum* were also identified [65]. Compared with these studies, in Nuomuhong Farm, Qinghai Province, the composition of pathogenic fungi causing root rot on Gouqi differed. In this study, *F. chlamydosporum*, *A. alternata*, *Ple. plurivora*, *Mortierella alpina*, and *Ple. cucumerina* were not found. We also identified *F. oxysporum*, *F. solani*, *F. tricinctum*, *F. culmorum*, *F. equiseti*, and *C. rosea*. This phenomenon might be related to regional differences and different planting years. Different planting areas exhibit considerable variation in ecological and climatic conditions, including altitude, annual mean temperature, and annual precipitation [66].

Ningxia and Gansu are temperate continental semi-arid climates, and Qinghai is the plateau continental climate [67]. The altitude of Gouqi root rot sampling sites in Gansu and Ningxia is between 1180 m and 1628 m [11]; however, the main cultivation area of Gouqi in Qinghai Province is Haixi Mongolian and Tibetan Autonomous Prefecture with an altitude of over 2700 m.

### 4.4. Biological Characteristics of Root Rot Pathogens from L. barbarum Differ from Those of the Pathogens from Other Hosts

Biological characteristics are important indicators of pathogens and can be used to predict the occurrence period and severity of plant diseases. Moreover, biological characteristics of plant pathogens are very important to pathogen research and disease control as the occurrence of plant diseases is not only influenced by external environmental conditions, but is also closely related to the inherent characteristics of the pathogens [68]. Therefore, the biological characteristics of 12 pathogens species, including the optimal pH, temperature, and carbon and nitrogen sources, were also determined in this study. Li et al. found that optimum growth conditions for *F. oxysporum*, causing black rot in *Gastrodia elata*, were as follows: pH 9, 28 °C, fructose as a carbon source, and beef extract as a nitrogen source. The strain died after 40 min of treatment at 60 °C [69]. Here, the optimum carbon source for *F. oxysporum* were sucrose, dextrin, soluble starch, and fructose. The optimum nitrogen sources were beef extract, peptone, and yeast powder. The lethal temperature was 65 °C, and the best pH was 10. The lethal temperature and best pH differed from those reported by Li et al. [69]. According to Li et al., the optimum growth conditions for *F. solani*, causing black rot in *Gastrodia elata*, were as follows: pH 7, 30 °C, mannitol as a carbon source, sodium nitrate as a nitrogen source, and PDA medium as a base medium. The strain died after 40 min of treatment at 65 °C [69]. In this study, the optimum culture medium for *F. solani* was PDA, and the lethal temperature was 65 °C. A pH of 10, a carbon source of mannitol, and a nitrogen source of sodium nitrate were optimal. Therefore, the results for *F. solani* are similar to those found by Li et al. [57]. Nikitin et al. found that *F. acuminatum* and *F. culmorum* were mostly identified in temperate climates and did not develop at temperatures above 25 °C [70]. Li et al. found that *F. sambucinum* and *F. culmorum*, causing potato dry rot, were sensitive to temperature and pH. *Fusarium sambucinum* showed favorable growth on the glucose-based culture, while *F. culmorum* preferred the sucrose-based culture [71]. Here, the lethal temperature of *F. acuminatum* was 50 °C, with the lethal temperature for *F. culmorum* being 50 °C, and the best carbon sources were sucrose, glucose, dextrin, and soluble starch. The biological characteristics of *F. acuminatum* and *F. culmorum* differed from those observed by Li et al. [71] and Nikitin et al. [70]. Zhang et al. found that *F. equiseti* from *A. membranaceus* could not grow below 5 °C or above 40 °C; the optimum pH value was 6, and the optimum nitrogen source was peptone [72]. In our study, we found the lethal temperatures of *F. equiseti* was 55 °C, and one of the optimum nitrogen sources was peptone. Tang et al. found that the temperature for *F. avenaceum* to be able to grow from *T. aestivum* was 15–25 °C, and the optimum carbon and nitrogen sources were soluble starch and peptone [73]. We found that the optimum carbon and nitrogen sources for *F. avenaceum* were soluble starch and yeast powder. These differences in the biological characteristics might be related to the source, hosts, environment of the isolates, and experimental environment.

In summary, *M. bolleyi*, *F. avenaceum*, and *F. citri*. are novel pathogens that caused *L. barbarum* root rot in China. The results of diversity and the biological characteristics of those pathogens in this study lay the foundation for determining the occurrence patterns of diseases and formulating prevention and control measures.

## 5. Conclusions

This study has provided preliminary insights into the fungal species responsible for root rot in *L. barbarum* at Nuomuhong Farm in Qinghai Province, China. Following Koch’s postulates, 12 pathogen species were identified to cause root rot in *L. barbarum*. The dominant pathogens included *F. equiseti*, *C. rosea*, and *M. bolleyi*. This research marks the first report of *M. bolleyi*, *F. avenaceum*, and *F. citri* as pathogens causing *L. barbarum* root rot in China. The lethal temperatures for these 12 pathogen species generally exceeded 50 °C. The best media for the 12 pathogens were PDA, CM, and CA. The optimum carbon sources were sucrose, dextrin, and soluble starch, and the optimum nitrogen sources were beef extract, peptone, and yeast extract. These results establish a foundation for the comprehensive prevention and control of Gouqi root rot. Future research will be focused on the screening and development of control agents for these 12 pathogenic fungi species, and the biological characteristics we found in this study will be used to develop methods to control root rot of Gouqi.

## Data Availability

The original contributions presented in this study are included in the article/Supplementary Material. Further inquiries can be directed to the corresponding author.

## Supplementary Materials

The following supporting information can be downloaded at: https://www.mdpi.com/article/10.3390/jof12010062/s1, Table S1. Conidia length and width (μm, presented as means and standard deviations) of twelve species of root rot pathogens from *L. barbarum*. Table S2. Effects of different media on the growth rate of root rot pathogens from *L. barbarum* (cm, presented as means and standard deviations, letters represented the significance of differences). Table S3. Effects of different temperatures on the growth rate of pathogens from *L. barbarum* (cm, presented as means and standard deviations, letters represented the significance of differences). Table S4. Effects of different pH on the growth rate of pathogens from *L. barbarum* (cm, presented as means and standard deviations, letters represented the significance of differences). Table S5. Effects of different carbon sources on the growth rate of pathogens from *L. barbarum* (cm, presented as means and standard deviations, letters represented the significance of differences). Table S6. Effects of different nitrogen sources on the growth rate of pathogens from *L. barbarum* (cm, presented as means and standard deviations, letters represented the significance of differences).
