# Supplementary material for: Diversity, Pathogenicity, and Biological Characteristics of Root Rot Pathogens from Lycium barbarum L. in Qinghai Province, China
Source: J Fungi (Basel). 2026 Jan 13;12(1):62. doi: 10.3390/jof12010062 (PMC12842772; doi:10.3390/jof12010062)
Supplement: Supplementary file 1 [file jof-12-00062-s001.zip › jof-3924579-supplementary.pdf]

Table S1. Conidia length and width (μm, presented as means and standard deviations) of twelve species of root rot pathogens from *L. barbarum*

Types of spores		*F. equiseti*	*C. rosea*	*M. bolleyi*	*F. solani*	*F. acuminatum*	*F. sambucinum*	*F. avenaceum*	*F. citri*	*F. incarnatum*	*F. culmorum*	*F. oxysporum*	*F. tricinctum*
conidia	microconidium	11.56±1.46×3.85±0.56	7.04±0.41×4.36±0.37	9.55±2.04×3.39±0.78	7.63±0.91×4.7±0.68	8.82±1.65×4.32±0.83	7.72±1.36×4.22±0.92	rare	rare	rare	8.24±1.56×4.43 ±1.14	7.22±1.12×3.26±0.70	13.20±2.16×4.52±1.44
	macroconidium	26.44±2.14×5.69±1.43			39.09±4.63×7.07±1.21	20.16±3.02×4.79±0.88	34.75±5.38×7.56±1.20	46.45±4.96×5.74 ±1.54	30.84±5.41×5.72±1.02	30.02±1.51×6.17±1.07	37.99±1.96×8.97±0.46	33.68±2.21×11.45±0.42	33.97±4.95×7.17±1.34
chlamydospore		16.34±1.58×14.15±2.08	11.38±2.25×12.45±2.02	11.28±1.46×8.00±0.59	15.74±2.02×14.19±1.90	13.07±2.15×12.84±1.23	13.12±3.80×9.45±2.15	not found	11.32±2.10×10.57±2.93	13.06±3.48×16.73±2.54	13.77±2.71×11.91±1.79	13.91±1.36×12.77±1.68	14.76±4.45×13.56±2.25

Table S2 Effects of different media on the growth rate of root rot pathogens from *L. barbarum* (cm, presented as means and standard deviations, letters represented the significance of differences).

**Media**	***F. equiseti***	***C. rosea***	***M. bolleyi***	***F. solani***	***F. acuminatum***	***F. sambucinum***	***F. avenaceum***	***F. citri***	***F. incarnatum***	***F. culmorum***	***F. oxysporum***	***F. tricinctum***
PDA	0.68±0.06b	0.44±0.04b	0.47±0.19bc	0.78±0.01a	0.52±0.01c	0.80±0.00a	0.80±0.01a	0.80±0.00ab	0.80±0.00a	0.80±0.00a	0.80±0.00a	0.72±0.08ab
CM	0.79±0.00a	0.50±0.01b	0.34±0.02c	0.77±0.01b	0.52±0.06c	0.80±0.01a	0.62±0.07b	0.80±0.01a	0.80±0.01a	0.80±0.00a	0.78±0.01c	0.64±0.09bc
CA	0.79±0.00a	0.50±0.07b	0.28±0.02c	0.72±0.01d	0.62±0.07b	0.80±0.00a	0.77±0.03a	0.79±0.01b	0.80±0.00a	0.80±0.00a	0.79±0.01b	0.68±0.07bc
MB	0.80±0.01a	0.61±0.04a	0.60±0.16ab	0.76±0.01b	0.53±0.01c	0.76±0.04a	0.56±0.06c	0.80±0.00ab	0.80±0.01a	0.80±0.00a	0.80±0.01a	0.61±0.05c
CLA	0.79±0.01a	0.48±0.03b	0.69±0.03a	0.73±0.00c	0.74±0.03a	0.78±0.03a	0.79±0.01a	0.80±0.01a	0.79±0.02b	0.80±0.00a	0.80±0.00a	0.80±0.01a

Table S3. Effects of different temperatures on the growth rate of pathogens from *L. barbarum* (cm, presented as means and standard deviations, letters represented the significance of differences).

**Temperature**	***F. equiseti***	***C. rosea***	***M. bolleyi***	***F. solani***	***F. acuminatum***	***F. sambucinum***	***F. avenaceum***	***F. citri***	***F. incarnatum***	***F. culmorum***	***F. oxysporum***	***F. tricinctum***
35℃	0.76±0.03a	0.51±0.05a	0.36±0.03a	0.77±0.01a	0.61±0.01a	0.79±0.01a	0.80±0.01a	0.79±0.01a	0.79±0.01a	0.80±0.01a	0.79±0.00a	0.79±0.01a
40℃	0.78±0.02a	0.51±0.01a	0.34±0.01a	0.78±0.01a	0.61±0.03a	0.79±0.01a	0.78±0.01a	0.80±0.01a	0.79±0.01a	0.79±0.01a	0.80±0.01a	0.76±0.04a
50℃	0.77±0.03a	0.40±0.03b	0.25±0.01b	0.77±0.01a	0	0	0.17±0.00b	0.58±0.12b	0.79±0.01a	0	0.80±0.01a	0.63±0.05b
55℃	0	0	0	0.47±0.00b	0	0	0	0	0	0	0.79±0.01a	0
60℃	0	0	0	0.35±0.00c	0	0	0	0	0	0	0.80±0.01a	0
65℃	0	0	0	0	0	0	0	0	0	0	0	0
70℃	0	0	0	0	0	0	0	0	0	0	0	0

Table S4. Effects of different Ph on the growth rate of pathogens from *L. barbarum* (cm, presented as means and standard deviations, Letters represented the significance of differences).

**pH value**	***F. equiseti***	***C. rosea***	***M. bolleyi***	***F. solani***	***F. acuminatum***	***F. sambucinum***	***F. avenaceum***	***F. citri***	***F. incarnatum***	***F. culmorum***	***F. oxysporum***	***F. tricinctum***
4	0.59±0.02f	0.50±0.03c	0.43±0.01b	0.68±0.01e	0.45±0.03d	0.79±0.01ab	0.52±0.01e	0.80±0.01a	0.78±0.01e	0.79±0.01ab	0.70±0.02c	0.67±0.04d
5	0.70±0.02cd	0.52±0.02abc	0.36±0.01cde	0.71±0.00c	0.57±0.01c	0.79±0.01ab	0.70±0.02cd	0.79±0.01ab	0.79±0.01cd	0.79±0.00b	0.79±0.00a	0.77±0.02abc
6	0.73±0.01abc	0.54±0.03ab	0.35±0.01e	0.73±0.00ab	0.63±0.03b	0.79±0.01ab	0.76±0.03b	0.79±0.01ab	0.79±0.01bc	0.79±0.00b	0.79±0.00a	0.78±0.01ab
7	0.75±0.01a	0.53±0.00ab	0.35±0.01de	0.70±0.01d	0.64±0.03b	0.79±0.00b	0.79±0.01ab	0.79±0.01ab	0.78±0.01de	0.79±0.00b	0.79±0.02a	0.79±0.01a
8	0.73±0.04ab	0.53±0.03ab	0.38±0.01c	0.72±0.01b	0.73±0.04a	0.80±0.01a	0.79±0.01ab	0.79±0.01ab	0.80±0.00a	0.79±0.00b	0.75±0.01b	0.78±0.01ab
9	0.73±0.02abc	0.54±0.02a	0.59±0.02a	0.73±0.01ab	0.58±0.02c	0.80±0.01a	0.76±0.03b	0.80±0.01a	0.80±0.00a	0.79±0.01ab	0.79±0.01a	0.78±0.01ab
10	0.66±0.03e	0.51±0.01bc	0.36±0.04cd	0.74±0.01a	0.59±0.03c	0.79±0.01ab	0.72±0.02c	0.79±0.00b	0.80±0.00a	0.80±0.01a	0.80±0.01a	0.74±0.05c

11	0.70±0.02bcd	0.52±0.01abc	0.36±0.02cde	0.73±0.00ab	0.58±0.02c	0.79±0.01ab	0.68±0.03d	0.79±0.00b	0.80±0.01ab	0.79±0.01ab	0.79±0.01a	0.75±0.02bc

Table S5. Effects of different carbon sources on the growth rate of pathogens from *L. barbarum* (cm, presented as means and standard deviations, Letters represented the significance of differences).

**Carbon source**	***F. equiseti***	***C. rosea***	***M. bolleyi***	***F. solani***	***F. acuminatum***	***F. sambucinum***	***F. avenaceum***	***F. citri***	***F. incarnatum***	***F.* culmorum**	***F. oxysporum***	***F. tricinctum***
CK	0.80±0.01ab	0.44±0.03cd	0.30±0.01c	0.78±0.01a	0.64±0.01b	0.75±0.02b	0.62±0.03cd	0.73±0.03bc	0.79±0.01ab	0.79±0.00a	0.79±0.01ab	0.65±0.02d
glucose	0.76±0.05b	0.43±0.02d	0.35±0.02b	0.79±0.01a	0.48±0.02d	0.72±0.06bc	0.59±0.03de	0.73±0.04bc	0.77±0.03b	0.80±0.00a	0.80±0.01a	0.74±0.03b
sucrose	0.80±0.00ab	0.43±0.01cd	0.63±0.02a	0.79±0.00a	0.46±0.02d	0.79±0.01a	0.64±0.05cd	0.80±0.00a	0.80±0.00a	0.80±0.00a	0.80±0.01a	0.79±0.01a
mannitol	0.80±0.01ab	0.45±0.03cd	0.37±0.20c	0.80±0.01a	0.39±0.02e	0.75±0.01b	0.46±0.03g	0.79±0.00a	0.79±0.01ab	0.79±0.01a	0.80±0.01a	0.71±0.06c
dextrin	0.79±0.01ab	0.48±0.07bc	0.30±0.03c	0.74±0.01c	0.52±0.01c	0.80±0.01a	0.75±0.04a	0.79±0.01a	0.80±0.01a	0.80±0.01a	0.80±0.01a	0.80±0.00a
maltose	0.70±0.05c	0.50±0.06b	0.29±0.02c	0.73±0.02c	0.66±0.05a	0.61±0.04d	0.69±0.02b	0.75±0.02bc	0.80±0.01a	0.79±0.01a	0.80±0.01a	0.72±0.02bc
soluble starch	0.79±0.01ab	0.43±0.03d	0.30±0.03c	0.76±0.02b	0.69±0.03a	0.80±0.01a	0.76±0.04a	0.79±0.01a	0.80±0.01a	0.80±0.01a	0.80±0.01a	0.79±0.01a

lactose	0.65±0.03d	0.44±0.08bcd	0.24±0.03de	0.73±0.01c	0.39±0.03e	0.71±0.01c	0.56±0.03ef	0.63±0.04d	0.64±0.02d	0.69±0.17b	0.78±0.01b	0.47±0.02f
cellulose	0.79±0.01ab	0.51±0.04b	0.37±0.02b	0.79±0.01a	0.72±0.03a	0.79±0.01a	0.70±0.03b	0.79±0.00a	0.17±0.02e	0.79±0.00a	0.79±0.01ab	0.78±0.01a
fructose	0.79±0.01ab	0.57±0.03a	0.25±0.02de	0.79±0.00a	0.53±0.02c	0.79±0.00a	0.58±0.02def	0.79±0.00a	0.78±0.01ab	0.79±0.00a	0.80±0.01a	0.70±0.02c
xylose	0.72±0.04c	0.44±0.02cd	0.21±0.03e	0.76±0.01b	0.46±0.04d	0.74±0.02bc	0.54±0.02f	0.72±0.01c	0.75±0.02c	0.79±0.01a	0.79±0.01ab	0.59±0.02e

Table S6. Effects of different nitrogen sources on the growth rate of pathogens from *L. barbarum* (cm, presented as means and standard deviations, Letters represented the significance of differences).

**Nitrogen source**	***F. equiseti***	***C. rosea***	***M. bolleyi***	***F. solani***	***F. acuminatum***	***F. sambucinum***	***F. avenaceum***	***F. citri***	***F. incarnatum***	***F. culmorum***	***F. oxysporum***	***F. tricinctum***
CK	0.80±0.00a	0.44±0.04bc	0.63±0.02c	0.71±0.02b	0.71±0.05a	0.69±0.03d	0.54±0.02c	0.62±0.08c	0.61±0.04e	0.80±0.01ab	0.74±0.02c	0.73±0.01b
beef extract	0.80±0.00a	0.43±0.01bc	0.80±0.00a	0.76±0.02a	0.57±0.10cd	0.80±0.00a	0.75±0.03a	0.71±0.00b	0.80±0.00a	0.80±0.00a	0.80±0.00a	0.80±0.01a
urea	0.80±0.01a	0.38±0.01cd	0.69±0.01b	0.64±0.01c	0.37±0.01f	0.71±0.02cd	0.49±0.08d	0.68±0.03b	0.72±0.04c	0.80±0.00a	0.79±0.01ab	0.49±0.02d
peptone	0.80±0.00a	0.51±0.10a	0.80±0.00a	0.72±0.02b	0.64±0.05bc	0.74±0.04bc	0.76±0.02a	0.78±0.04a	0.80±0.00a	0.80±0.00a	0.80±0.01ab	0.80±0.01a
yeast powder	0.80±0.01a	0.45±0.01ab	0.80±0.00a	0.71±0.01b	0.59±0.06bc	0.80±0.01a	0.78±0.02a	0.80±0.00a	0.80±0.00a	0.80±0.01ab	0.80±0.00a	0.80±0.00a
sodium nitrate	0.79±0.01a	0.42±0.02bc	0.25±0.01f	0.79±0.01a	0.51±0.02d	0.77±0.02ab	0.69±0.04b	0.79±0.01a	0.78±0.01ab	0.80±0.00a	0.79±0.01ab	0.73±0.04b
ammonium chloride	0.64±0.05b	0.28±0.04e	0.59±0.05d	0.49±0.05e	0.35±0.05f	0.53±0.07e	0.34±0.04e	0.42±0.03d	0.50±0.04f	0.78±0.03c	0.67±0.03d	0.45±0.05e

ammonium nitrate	0.79±0.00a	0.35±0.03d	0.71±0.03b	0.60±0.02d	0.44±0.01e	0.78±0.01ab	0.48±0.04d	0.63±0.06c	0.65±0.02d	0.79±0.01abc	0.78±0.01b	0.65±0.03c
saltpeter	0.80±0.01a	0.51±0.06a	0.34±0.01e	0.78±0.02a	0.52±0.01d	0.78±0.01ab	0.66±0.06b	0.73±0.03b	0.76±0.03b	0.79±0.00abc	0.78±0.01b	0.79±0.01a
